# The association between the use of shift schedule evaluation tool with ergonomics recommendations and occupational injuries: A 4-year prospective cohort study among healthcare workers

**DOI:** 10.5271/sjweh.4068

**Published:** 2023-02-27

**Authors:** Rahman Shiri, Jarno Turunen, Kati Karhula, Aki Koskinen, Mikael Sallinen, Annina Ropponen, Jenni Ervasti, Mikko Härmä

**Affiliations:** 1 Finnish Institute of Occupational Health, Helsinki, Finland; 2 Karolinska Institute, CNS, Division of Insurance Medicine, Stockholm, Sweden

**Keywords:** accidental fall, accident, injury, occupational safety, shift work schedule, shift worker, sprain, strain, workplace, wound

## Abstract

**Objective:**

This study aimed to find out whether utilizing a shift schedule evaluation tool with ergonomics recommendations for working hours has favorable effects on the incidence of occupational injuries.

**Methods:**

This 4-year prospective cohort study (2015–2018) consisted of a dynamic cohort of healthcare shift workers (N=29 237) from ten hospital districts and six cities in Finland. Working hour characteristics and occupational injuries were measured with daily registry data. Multilevel generalized linear model was used for the analyses, and the estimates were controlled for hierarchical structure of the data and confounders.

**Results:**

Ward heads of the cities used the shift schedule evaluation tool 3.2 times more often than ward heads of the hospital districts. Overall incidence of workplace and commuting injuries did not differ between users and non-users of the evaluation tool. The incidence of dislocations, sprains, and strains was lower in the users than non-users [adjusted odds ratio (OR) 0.88, 95% confidence interval (CI) 0.78–0.99]. Approximately 13% of this association was mediated by increase in realized shift wishes and 10% by increase in single days off. In subgroup analyses, the incidence of workplace injury (OR 0.83, 95% CI 0.69–0.99), and among types of injuries, the incidence of dislocations, sprains, and strains (OR 0.69, 95% CI 0.55–0.85) and falling, slipping, tripping, or overturning (OR 0.75, 95% CI 0.58–0.99) were lower in users than non-users among employees of the cities, but no association was found among employees of the hospital districts.

**Conclusion:**

The use of ergonomics recommendations for working hours is associated with a reduced risk of occupational injuries.

Occupational injuries are common among healthcare workers (1–5). Healthcare workers are at increased risk of occupational injuries due to patient care and clinical operations, individual and organizational risk factors (6). The incidence of occupational injuries ranges between 2% and 14% (1–5). Common occupational injuries include needlestick injuries (4, 7, 8), being cut by a sharp object and superficial injuries (1, 4, 9), sprains and strains (1, 9), and falling and slipping (3). Bone fractures (1, 9), violence (4) and psychological strain (1) are less common.

Previous studies have investigated the associations of sleep problems and working hour characteristics with occupational injuries. Some studies have found higher rates of occupational injuries among workers with sleep disturbance compared to those without sleep problems (9, 10). However, earlier research has reported inconsistent results on the association between short sleep and occupational injuries. A prospective cohort study (9) found an increased rate of occupational injuries among workers with short sleep, while a case–control (10) and crossover study (11) did not find an association between sleep duration and occupational injuries. Waking up too early, having difficulty falling asleep and nonrestorative sleep have been associated with commuting injuries (9). A study (10) found that sleep quality was associated with occupational injuries among employees with sleep duration of <7 hours or weekly working hours of >50 hours but not among employees with sleep duration of >7 hours or weekly working hours of <50 hours.

Long weekly working hours have been associated with insomnia and waking up with fatigue (12), and fatigue has been associated with occupational injuries (13). Some studies have found the associations of long weekly working hours (3, 14), long daily working hours (15, 16), or working mandatory or unplanned overtime (17), long shift (18), shift work (17–20), and short recovery (<11 hours) (21) with occupational injuries, but some other studies have not found the associations of long weekly working hours (10, 17, 19, 22), shift length (17), or short recovery (<11 hours) (18, 22) with occupational injuries. Long weekly working hours have been associated with the incidence of occupational injuries only among employees with insufficient rest breaks (14). Long working hours have not associated with mortality due to injuries (23).

A prospective cohort study (24) found that control over taking breaks or paid leave, and control over doing private errands during work were associated with a lower incidence of injuries, but control over starting and finishing times of daily work, and control over shift length did not influence the incidence of injuries. About 5% of the association between work time control and the incidence of injuries was mediated by sleep disturbance, but short sleep duration did not mediate the association (24). Our earlier study showed that using a shift schedule evaluation tool, which included ergonomic shift design recommendations, was associated with decreases in the number of consecutive workdays, >4 consecutive night shifts, night shift of ≥10 hours, and proportion of <11-hour shift intervals (25). Furthermore, the use of the shift schedule evaluation tool with ergonomics recommendations (hereafter referred to as the shift schedule evaluation tool) was associated with increase in the proportion of single days off (25).

A Finnish crossover study conducted among healthcare workers found increased risk of occupational injuries following ≥3 evening shifts, during workdays following night shifts and during long work shifts (18). The number of night shifts, length of the weekly working hours and short shift intervals (<11 hours) were not associated with occupational injuries (18). The aim of the current study was to assess whether using the shift schedule evaluation tool as part of the regular shift scheduling process is associated with the incidence of different types and causes of occupational injuries occurring at workplace or during commuting. Moreover, we examined whether the association differs between municipal and hospital healthcare workers or between younger and older healthcare workers. The possible mediation of the different working hour characteristics in the association between the use of shift schedule evaluation tool and occupational injuries was investigated based on the earlier methodological papers on the main dimension of working hour characteristics for epidemiologic studies, and the given recommendations (25, 26) (figure 1).

**Figure 1:**
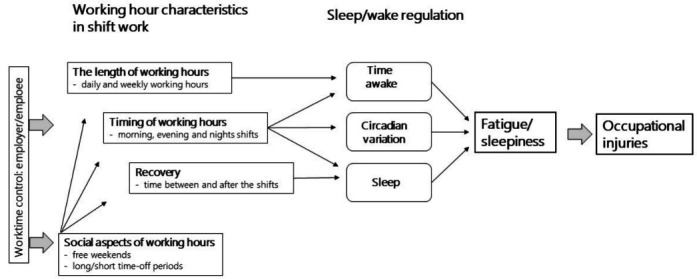
A schematic figure on the possible pathways from working hour characteristics in shift work to occupational injuries.

## Methods

### Population

This study is part of the ongoing Working Hours in the Finnish Public Sector (WHFPS) study (26). The study population consisted of a dynamic cohort of the healthcare employees who worked in one of the 10 hospital districts or 11 cities and used Titania® shift scheduling software. The details of the study population were described previously (25). Hospital districts cover specialized outpatient and inpatient health services. The healthcare of the cities covers primary healthcare at health centers, hospitals for elderly and chronically ill citizens needing 24/7 care, and home care for disabled citizens.

There were altogether 542 636 observations with annual working hour data between 2015 and 2018 (135 659 participants). Of those, 391 515 observations with missing data on the use of the shift schedule evaluation tool, 3264 observations with missing data on type of work, 31 635 observations of administrative employees, day workers and physicians, 8059 observations of employees with less than 31 work shifts within a year, and 58 observations with missing data on occupational injuries were excluded. The final sample of this open cohort included 108 105 observations in the 16 organizations (10 hospital districts and 6 cities): 24 186 healthcare shift workers in 2015 (table 1), 26 369 in 2016, 28 313 in 2017 and 29 237 shift workers in 2018. Payroll-based daily working hour data on planned and executed daily working hours were used to calculate working hour characteristics (25, 26). The information on the use of the shift schedule evaluation tool by the ward heads being responsible for shift scheduling was recorded in minutes and the minute-based use of the tool during all 3-week shift scheduling periods of the years 2015, 2016, 2017 and 2018 was used. When the shift schedule evaluation tool was used by the ward head, the employees of that ward were included in the intervention group. The employees working in the wards where the ward head did not use the shift schedule evaluation tool remained in the control group. However, in accordance with stepped wedge design, almost all the participants eventually started the use of the tool.

**Table 1:** The characteristics of the study population at baseline in 2015 (N=24 186). [SD=standard deviation.]

Characteristic	N	%	Mean	SD
Sex				
Women	20 971	86.7		
Men	3215	13.3		
Age (years)				
<30	5478	22.6		
30–39	6182	25.6		
40–49	5345	22.1		
50–59	5735	23.7		
>60	1446	6.0		
Organizations				
Hospital districts	13 289	54.9		
Cities	10 897	45.1		
Shift characteristics				
Night shifts (yes/no)	14 244	58.9		
Night shifts >12 hours among night shift workers (yes/no)	5755	40.4		
Consecutive night shifts >4 hours among night shift workers (yes/no)	2352	16.5		
% of working weeks >40 hours			25.9	13.4
% of working weeks >48 hours			5.3	6.9
% of shifts >12 hours			4.4	9.5
% of shift intervals <11 hours			15.0	12.0
% of night shifts >8 hours among night shift workers			24.2	20.3
% of night shifts >10 hours among night shift workers			18.5	19.3
% of night shifts >12 hours among night shift workers			1.6	5.5

It should be noted that the software saves information on the use of the evaluation tool only if changes were made to the schedule during the same session of the evaluation. If there were no need for changes in work schedule, no information was saved on the evaluation even if it actually took place. Since the use of the tool and recommendations included to the tool are quickly learned, we have reasons to assume that the positive number of minutes indicate that the recommendations are used and discontinuation of using the tool does not indicate that the intervention is no longer in effect (25).

### Outcomes

Occupational injuries were categorized as (i) workplace injuries and (ii) commuting injuries, as defined by the Workers’ Compensation Center using the Finnish legislation (the Workers’ Compensation Act 459/2015). In Finland, workplace and commuting injuries are compensated by a statutory insurance system. Data on workplace and commuting injuries were obtained from the Workers’ Compensation Center. Leisure-time injuries were excluded due to being incompletely registered by the insurance-based register. The types of workplace or commuting injuries with enough events for a multivariable analysis (27) were (i) dislocations, sprains, and strains, (ii) wounds and superficial injuries, and (iii) bone fractures. The causes of workplace or commuting injuries with sufficient events were (i) sudden physical or mental strain including noise and radiation, (ii) impact of a fixed surface or immobile cause (eg, falling), (iii) cutting caused by a sharp or rough object, (iv) animal or human bite, kick, etc. (used as an indicator of physical violence), (v) a moving agent (including pressurized liquids and gases), and (vi) compression or bruising. Common commuting injuries with sufficient events for a multivariable analysis were (i) falling, slipping, tripping, or overturning, and (ii) collision with a car, motorcycle, moped or bicycle. The number of events for different body locations were not sufficient to perform a multivariable analysis.

### Statistical analysis

This 4-year prospective cohort study was analyzed like a stepped wedge randomized controlled trial design (28) to investigate the association between the use of the shift schedule evaluation tool and the incidence of workplace and commuting injuries. For each year (2015, 2016, 2017 and 2018), the users of the shift schedule evaluation tool were defined as the intervention group and non-users of the shift schedule evaluation tool during the same year as the control group. The incidence of injuries among the users were compared with those of non-users. We used intention-to-treat principle and defined the intervention as using the shift schedule evaluation tool within the shift scheduling tool for at least one 3-week period. The use and recommendations included in the shift schedule evaluation tool are easy to learn, so discontinuing the use of the tool does not indicate that the recommendations are no longer used. Thus, following a stepped wedge clinical trial design, employees who used the shift schedule evaluation tool during at least one 3-week scheduling period were included in the intervention group for the upcoming years irrespective of the amount of future use of the shift schedule evaluation tool. Multi-level generalized linear model (GLM) with a binomial distribution and a logit link function was used to control for hierarchical structure of the data (individuals nested within units and units nested within organizations). The estimates were controlled for age, sex, number of contract days, number of night shifts and the use of participatory shift scheduling software. Subgroup analyses were performed for two age groups (<40 versus >40 years) based on median age, and two employer groups (hospital districts versus cities). The study did not have sufficient statistical power to split the sample into more age groups. A sensitivity analysis was conducted, and the follow-up ended in 2017 to improve balance in the number of observations in the intervention and control groups.

Mediation was tested using ‘medsem’ package in Stata version 17 (StataCorp LLC, College Station, TX, USA) and both Baron & Kenny approach modified by Iacobucci et al (29) and Zhao et al approach (30) were used. The working hour characteristics were selected for mediation based on our earlier study (25). The following continuous working hour characteristics measured annually between 2015–2018 were used for testing the mediation between the use of the shift schedule evaluation tool and the occupational injuries: realized shift wishes (based on the comparison of the final and draft shift schedules with information on which shifts/free days were wished by the employees), proportion of single days off, proportion of short shift intervals (<11 hours), number of consecutive workdays, number of >4 consecutive night shifts, proportion of long night shifts >12 hours, and proportion of short (<28 hours) recovery periods after the last night shifts.

## Results

Mean age of the study population at baseline in 2015 was 40.9 ± 12.1 years, and 48% were younger than 40 years and 87% were women (Table 1). Of the study population at baseline, 55% worked in the hospital districts and 45% worked in the cities, and 59% worked at least one night shift. On average, 26% of the working weeks were longer than 40 hours and 15% of the shift intervals were shorter than 11 hours. Of night shift workers, 40% had at least one night shift >12 hours during 2015, and 17% had at least once >4 consecutive night shifts.

The cumulative proportions of employees whose shift schedules were evaluated using the shift schedule evaluation tool by the ward heads for at least one 3-week period within a year were 45% in 2015, 62% in 2016, 77% in 2017, and 88% in 2018. On average, the shift schedule evaluation tool took mean 52 [standard deviation (SD) 241 (range 0–3937)] minutes for each ward head per year. Time used for the evaluation of the shift schedules in the cities was 3.2 times higher than in the hospital districts (mean 79, SD 304, versus mean 25, SD 144, minutes).

The incidence (per 100 person-years) of workplace injury was 2.5% and that of commuting injury was 1.3% during the follow-up. The incidence of workplace injury did not differ between employees of the hospital districts and the cities (2.49 versus 2.50%) or between employees <40 and ≥40 years (2.52 versus 2.47%). The incidence of commuting injury was higher among employees of the cities than hospital districts (1.4% versus 1.2%, P=0.030). The incidence of commuting injury did not differ between age groups. The annual incidence rates of workplace and commuting injuries did not change during the 4-year follow-up period.

### The association between the use of shift schedule evaluation tool and occupational injuries

Neither the overall incidence of workplace injuries nor that of commuting injuries differed between the users and non-users of the shift schedule evaluation tool. Of common types of injuries, the incidence of dislocations, sprains, and strains was lower among the users than non-users of the shift schedule evaluation tool after adjustment for covariates [odds ratio (OR) 0.88, 95% CI 0.78–0.99, table 2]. However, the incidence of wounds and superficial injuries and bone fractures did not differ between the users and non-users. Of causes of injuries, the incidence of sudden physical or mental strain including noise and radiation was lower in the users than non-users (OR 0.85, 95% CI 0.70–1.03), however, the difference was borderline statistically significant (P=0.097). The incidence of other causes of workplace injuries and common commuting injuries did not differ between the users and non-users (table 2).

**Table 2:** Difference in workplace and commuting injuries according to the use (no/yes) of the shift schedule evaluation tool. [OR=odds ratio; CI=confidence interval.]

Injury	No (N=33 566)		Yes (N=74 539)		Model I ^a^		Model II ^b^	
			
	Events	% of injury	Events	% of injury	OR	95% CI	OR	95% CI
Injuries								
Workplace or commuting	1283	3.82	2819	3.78	0.95	0.89–1.02	0.95	0.88–1.02
Workplace	864	2.57	1833	2.46	0.94	0.86–1.03	0.94	0.85–1.03
Commuting	419	1.25	986	1.32	0.99	0.86–1.12	1.00	0.87–1.14
Types of injury								
Dislocations, sprains, and strains	563	1.68	1158	1.55	0.88	0.79–0.99	0.88	0.78–0.99
Wounds and superficial injuries	442	1.32	939	1.26	0.97	0.86–1.10	0.97	0.85–1.10
Bone fractures	47	0.14	121	0.16	1.12	0.77–1.62	1.09	0.74–1.60
Causes of injury								
Sudden physical or mental strain (including noise and radiation)	212	0.63	398	0.53	0.84	0.70–1.01	0.85	0.70–1.03
Impact of a fixed surface or immobile cause (eg, falling)	188	0.56	396	0.53	0.92	0.75–1.11	0.93	0.76–1.14
Cutting caused by a sharp or rough object	135	0.40	308	0.41	1.07	0.85–1.33	1.07	0.85–1.36
Physical violence (animal or human bite, kick, etc.)	149	0.44	287	0.39	0.85	0.68–1.07	0.83	0.65–1.05
A moving agent (including pressurized liquids and gases)	49	0.15	148	0.20	1.39	0.97–1.99	1.35	0.92–1.96
Compression or bruising	37	0.11	103	0.14	1.15	0.74–1.78	1.16	0.74–1.80
Common commuting injuries								
Falling, slipping, tripping, or overturning	318	0.95	761	1.02	0.99	0.85–1.16	1.00	0.86–1.17
Collision with a car, motorcycle, moped or bicycle	39	0.12	107	0.14	1.19	0.79–1.78	1.23	0.82–1.85

a Adjusted for hierarchical structure of the data.

b Model I further adjusted for age, sex, number of days of work contract, number of night shifts, and the use of participatory shift scheduling software.

In subgroup analyses, the incidence of any workplace or commuting injury (OR 0.83, 95% CI 0.72–0.95), workplace injury (OR 0.83, 95% CI 0.69–0.99), dislocations, sprains, and strains (OR 0.69, 95% CI 0.55–0.85), falling, slipping, tripping, or overturning (OR 0.75, 95% CI 0.58–0.99), commuting injury (OR 0.81, 95% CI 0.64–1.03), and sudden physical or mental strain including noise and radiation (OR 0.71, 95% CI 0.50–1.02) were lower in the users than non-users of the shift schedule evaluation tool among employees of the cities (table 3). Among employees of the hospital districts, there were no differences in injuries, types and causes of injuries between the users and non-users of the shift schedule evaluation tool.

**Table 3:** Difference in workplace and commuting injuries according to the use (no/yes) of the shift schedule evaluation tool among the hospital districts and cities. [OR=odds ratio; CI=confidence interval.]

Injury	No		Yes		Model I ^a^		Model II ^b^	
			
	Events	% of injury	Events	% of injury	OR	95% CI	OR	95% CI
Cities (5934 non-users and 37 789 users)								
Injuries								
Workplace or commuting	269	4.53	1430	3.78	0.85	0.74–0.97	0.83	0.72–0.95
Workplace	168	2.83	923	2.44	0.85	0.71–1.01	0.83	0.69–0.99
Commuting	101	1.70	507	1.34	0.81	0.65–1.02	0.81	0.64–1.03
Types of injury								
Dislocations, sprains, and strains	128	2.16	586	1.55	0.72	0.58–0.89	0.69	0.55–0.85
Wounds and superficial injuries	78	1.31	462	1.22	0.93	0.74–1.16	0.91	0.71–1.15
Bone fractures	11	0.19	64	0.17	0.94	0.51–1.75	0.94	0.51–1.75
Causes of injury								
Sudden physical or mental strain (including noise and radiation)	43	0.72	198	0.52	0.74	0.53–1.04	0.71	0.50–1.02
Impact of a fixed surface or immobile cause (eg, falling)	37	0.62	211	0.56	0.87	0.58–1.29	0.84	0.55–1.27
Cutting caused by a sharp or rough object	17	0.29	159	0.42	1.48	0.90–2.42	1.55	0.91–2.64
Physical violence (animal or human bite, kick, etc.)	28	0.47	143	0.38	0.77	0.49–1.21	0.71	0.46–1.12
A moving agent (including pressurized liquids and gases)	10	0.17	69	0.18	1.09	0.59–2.02	0.95	0.50–1.82
Compression or bruising	10	0.17	57	0.15	0.87	0.44–1.72	0.91	0.45–1.82
Common commuting injuries								
Falling, slipping, tripping, or overturning	83	1.40	396	1.05	0.77	0.59–0.99	0.75	0.58–0.99
Collision with a car, motorcycle, moped or bicycle	9	0.15	56	0.15	0.98	0.51–1.88	1.17	0.59–2.33
Hospital districts (27 632 non-users and 36 750 users)								
Injuries								
Workplace or commuting	1014	3.67	1389	3.78	1.00	0.92–1.07	1.00	0.92–1.08
Workplace	696	2.52	910	2.48	0.98	0.88–1.09	0.98	0.88–1.09
Commuting	318	1.15	479	1.30	1.07	0.92–1.26	1.08	0.92–1.27
Types of injury								
Dislocations, sprains, and strains	435	1.57	572	1.56	0.96	0.84–1.09	0.96	0.84–1.10
Wounds and superficial injuries	364	1.32	477	1.30	0.98	0.85–1.14	0.98	0.85–1.13
Bone fractures	36	0.13	57	0.16	1.20	0.77–1.86	1.15	0.72–1.85
Causes of injury								
Sudden physical or mental strain (including noise and radiation)	169	0.61	200	0.54	0.88	0.71–1.09	0.90	0.72–1.13
Impact of a fixed surface or immobile cause (eg, falling)	151	0.55	185	0.50	0.94	0.75–1.17	0.96	0.76–1.22
Cutting caused by a sharp or rough object	118	0.43	149	0.41	0.97	0.74–1.26	0.96	0.73–1.26
Physical violence (animal or human bite, kick, etc.)	121	0.44	144	0.39	0.88	0.68–1.14	0.87	0.67–1.14
A moving agent (including pressurized liquids and gases)	39	0.14	79	0.21	1.50	0.98–2.27	1.49	0.95–2.33
Compression or bruising	27	0.10	46	0.13	1.31	0.77–2.22	1.32	0.77–2.27
Common commuting injuries								
Falling, slipping, tripping, or overturning	235	0.85	365	0.99	1.12	0.93–1.34	1.13	0.94–1.35
Collision with a car, motorcycle, moped or bicycle	30	0.11	51	0.14	1.26	0.77–2.05	1.22	0.75–1.98

a Adjusted for hierarchical structure of the data.

b Model I further adjusted for age, sex, number of days of work contract, number of night shifts, and the use of participatory shift scheduling software.

In age stratified analysis, the incidence of dislocations, sprains, and strains was lower in the users than non-users of the shift schedule evaluation tool (OR 0.82, 95% CI 0.69–0.97) among employees aged <40 years (supplementary material, www.sjweh.fi/article/4068, table S1). Among employees aged ≥40 years, the incidence of wound and superficial injuries (OR 0.81, 95% CI 0.67–0.98), workplace/commuting injuries (OR 0.93, 95% CI 0.85–1.01) and workplace injuries (OR 0.89, 95% CI 0.78–1.02) were lower in users than non-users.

The results of a sensitivity analysis are reported in supplementary table S2. After limiting follow-up to 2015–2017, the incidence of workplace/commuting injuries (OR 0.84, 95% CI 0.72–0.98) and dislocations, sprains, and strains (OR 0.71, 95% CI 0.57–0.89) was lower in users than non-users of the shift schedule evaluation tool among employees of the cities. The incidence of physical violence (animal or human bite, kick, etc.) was lower in the users than non-users of the shift schedule evaluation tool among total sample (OR 0.73, 95% CI 0.55–0.96) and among employees of the hospital districts (OR 0.72, 95% CI 0.53–0.98).

### Mediation

There was indirect-only mediation (full mediation) for the association between the use of shift schedule evaluation tool and the incidence of dislocations, sprains, and strains. Approximately 13% of the association between the use of shift schedule evaluation tool and dislocations, sprains, and strains was mediated by increase in realized shift wishes (P=0.008 for the Sobel’s test and P=0.009 for the Monte Carlo test), and 10% was mediated by increase in the proportion of single days off (P=0.009 for the Sobel’s test and P=0.010 for the Monte Carlo test).

Among employees of the cities, 5% of the association between the use of shift schedule evaluation tool and workplace injuries was mediated by decrease in the number of consecutive night shifts (P=0.058 for the Sobel’s test and P=0.059 for the Monte Carlo test) and 5% of the association between the use of shift schedule evaluation tool and falling, slipping, tripping, or overturning was mediated by increase in realized shift wishes (P=0.054 for the Sobel’s test and P=0.060 for the Monte Carlo test). None of the working hour characteristics mediated the association between the use of shift schedule evaluation tool and workplace/commuting injury, or dislocations, sprains, and strains among employees of the cities.

In the age group <40 years, about 6% of the association between the use of shift schedule evaluation tool and dislocations, sprains, and strains was mediated by increase in realized shift wishes (P=0.043 for the Sobel’s test and P=0.047 for the Monte Carlo test), 6% by decrease in the proportion of shift intervals <11 hours (P=0.045 for the Sobel’s test and P=0.046 for the Monte Carlo test), 13% by decrease in the proportion of short recovery periods (<28 hours) after the last night shifts (P=0.086 for the Sobel’s test and P=0.089 for the Monte Carlo test), and 5% was mediated by decrease in the number of consecutive workdays (P=0.078 for the Sobel’s test and P=0.080 for the Monte Carlo test).

In the age group ≥40 years, about 6% of the association between the use of shift schedule evaluation tool and wound and superficial injuries was mediated by increase in the proportion of single days off (P=0.011 for the Sobel’s test and P=0.011 for the Monte Carlo test), and 4% was mediated by decrease in the proportion of short recovery periods after the last night shifts <28h (P=0.064 for the Sobel’s test and P=0.068 for the Monte Carlo test).

## Discussion

The findings of this study indicate that the use of the shift schedule evaluation tool with ergonomic shift design recommendations increased the proportions of realized shift wishes and single days off which, in turn, decreased the risk of dislocations, sprains, and strains. In employees <40 years, the use of the shift schedule evaluation tool decreased short shift intervals, short recovery periods after the last night shifts, and the number of consecutive workdays which in turn decreased the risk of dislocations, sprains, and strains.

Among employees of the cities, the use of the recommendations for good shift ergonomics was associated with more beneficial effects on occupational injuries than among employees of the hospital districts. The ward heads of the cities spent over 3-fold more time on using the shift schedule evaluation tool for the schedule design and shift wishes than the ward heads of the hospital districts, that is likely to explain the more beneficial effects of the use of the evaluation tool in cities. Our earlier research among the partially same study population (25) showed stronger associations between the use of the shift schedule evaluation tool and the recommended working hour characteristics among employees of the cities than among employees of the hospital districts. Furthermore, the difference in the observed association between employees of the cities and hospital districts may be due to better implementation of the recommendations for good shift ergonomics among employees of the cities than among employees of the hospital districts. Among employees of the cities, 70% started to use the shift schedule evaluation tool in 2015 and 99% used it for at least one 3-week period by 2018, while among employees of the hospital districts, only 24% used the shift schedule evaluation tool in 2015 and by 2018 the proportion of the users reached 82%.

The cities are responsible for providing services at health centers, hospitals for elderly and chronically ill citizens, and home care for disabled citizens, whereas hospital districts provide specialized outpatient and inpatient health services. Thus, differences in work characteristics may also partly explain the observed differences in occupational injuries. For instance, the use of the shift schedule evaluation tool was associated with lower rate of night shift ≥10 hours among the employees of the cities only (25).

A previous study from the same cohort showed that the frequent use of the shift schedule evaluation tool reduced the number of consecutive workdays, >4 consecutive night shifts, night shift of ≥10 hours, and proportion of short shift intervals (of <11 hours) and increased the proportion of single days off increasing recovery (25). Similar significant associations between frequent use of the shift schedule evaluation tool and the above-mentioned working hour characteristics were also found among the current study population. The decrease of night work and the increase of recovery between and after the shifts can probably reduce fatigue and improve sleep quality (31). The level of fatigue has been higher among nurses who work weekly overtime (32), long shifts (32, 33), more or consecutive night shifts (31, 33), or have short shift intervals (31). Working weekly overtime (12), consecutive night shift (31), and short shift intervals (31) are also associated with sleep disturbance. Fatigue (11, 13, 34), sleep disturbance (9, 10), weekly overtime (35), night shifts (20, 36), long shift length (18, 36, 37) and short recovery (<11 hours) (21) have been recognized as risk factors for occupational injuries.

The strengths of the current study were associated with the recruitment of a large sample of healthcare shift workers from both municipal health care sector and hospitals. Both the use of the shift schedule evaluation tool and working hour characteristics were objectively measured. We defined the intervention as using the shift schedule evaluation tool for at least one 3-week period according to intention-to-treat principle to include only objectively verified as never users of the evaluation tool in the control group. The current study has some limitations. The data were based on payroll reports and the unmeasured confounding cannot be ruled out. The users and nonusers of the shift schedule evaluation tool may have differed in the rates of sleep problems, lifestyle habits, and psychosocial risk factors. Employees who worked >40 hours per week were more motivated to use the shift schedule evaluation tool than employees who worked <40 hours per week (25). This suggests that the rates of fatigue and sleep disturbance as the determinants of occupation injuries might have differed between the users and nonusers of the shift schedule evaluation tool. In the current study, workplace injuries also included injuries caused by physical violence such as human bite or kick indicating work-related violence. The use of the shift schedule evaluation tool was not associated with physical violence rate among total sample (2015–2018 follow-up), and the exclusion of 436 cases of animal or human bite, kick, etc. from workplace injuries slightly attenuated the observed associations.

### Concluding remarks

The use of the shift schedule evaluation tool was associated with reduced risk of occupational injuries among healthcare shift workers of the cities. However, it was not associated with occupational injuries among healthcare shift workers of the hospital districts with shorter average time used for the evaluation of the schedules. The association was indirect and mediated mostly by improved chances of recovery between and after the shifts. In age-specific analysis, the use of the shift schedule evaluation tool was associated with reduced risk of dislocations, sprains, and strains in employees <40 years and with reduced risk of wound and superficial injuries in employees aged ≥40 years.

### Ethics approval

This study utilized register data of working hours and occupational injuries. According to Finnish legislation, using register data for public interest research does not require ethics approval.

### Conflict of interest

The authors declare no conflicts of interest.

### Funding

This study was funded by the Finnish Work Environment Fund (no 210064) and the European Union’s Horizon 2020 research and innovation programme (no 826266).

## Supplementary material

Supplementary material
