# Supplementary material for: The association between the use of shift schedule evaluation tool with ergonomics recommendations and occupational injuries: A 4-year prospective cohort study among healthcare workers
Source: Scand J Work Environ Health. 2023 Feb 27;49(2):108–16. doi: 10.5271/sjweh.4068 (PMC10577015; doi:10.5271/sjweh.4068)
Supplement: Supplementary material [file SJWEH-49-108-S001.pdf]

The association between the use of shift schedule evaluation tool with ergonomics recommendations and occupational injuries: A 4-year prospective cohort study among healthcare workers[1]

by Rahman Shiri, MD, PhD,[2] Jarno Turunen, MSc, Kati Karhula, PhD, Aki Koskinen, MSc, Mikael Sallinen, PhD, Annina -Ropponen, PhD, Jenni Ervasti, PhD, Mikko Härmä, MD, PhD

1. Supplementary material
2. Correspondence to: Rahman Shiri, Finnish Institute of Occupational Health, P.O. Box 18,FI-00032 Työterveyslaitos, Helsinki. [E-mail: rahman.shiri@ttl.fi]

Supplementary table S1: Difference in workplace and commuting injuries according to the use (no/yes) of the shift schedule evaluation tool among employees aged <40 years and those aged 40 years or older

Injury	No		Yes		Model I		Model II	
	Events	% of injury	Events	% of injury	OR	95% CI	OR	95% CI
**Aged <40 years** (17,347 non-users and 39,167 users)								
Injuries								
Workplace or commuting	657	3.79	1500	3.83	0.96	0.87-1.06	0.96	0.86-1.06
Workplace	439	2.53	986	2.52	0.97	0.85-1.10	0.97	0.85-1.10
Commuting	218	1.26	514	1.31	0.98	0.81-1.18	0.98	0.81-1.18
Types of injury								
Dislocations, sprains, and strains	302	1.74	620	1.58	0.84	0.71-0.99	0.82	0.69-0.97
Wounds and superficial injuries	215	1.24	506	1.29	1.10	0.92-1.30	1.11	0.93-1.32
Bone fractures	23	0.13	65	0.17	1.23	0.72-2.10	1.23	0.71-2.14
Causes of injury								
Sudden physical or mental strain (including noise and radiation)	113	0.65	227	0.58	0.82	0.64-1.06	0.84	0.64-1.10
Impact of a fixed surface or immobile cause (e.g., falling)	93	0.54	194	0.50	0.90	0.69-1.18	0.93	0.71-1.23
Cutting caused by a sharp or rough object	70	0.40	174	0.44	1.22	0.92-1.61	1.25	0.94-1.67
Physical violence (animal or human bite, kick, etc.)	74	0.43	154	0.39	0.92	0.67-1.25	0.88	0.64-1.21
A moving agent (including pressurized liquids and gases)	28	0.16	84	0.21	1.34	0.82-2.21	1.32	0.78-2.23
Compression or bruising	12	0.07	46	0.12	1.57	0.78-3.18	1.62	0.79-3.30
Common commuting injuries								
Falling, slipping, tripping, or overturning	161	0.93	401	1.02	1.01	0.80-1.26	1.00	0.79-1.25
Collision with a car, motorcycle, moped or bicycle	17	0.10	60	0.15	1.67	0.94-2.94	1.71	0.95-3.08
**Aged 40 years or older** (16,219 non-users and 35,372 users)								

Injury	No		Yes		Model I		Model II	
	Events	% of injury	Events	% of injury	OR	95% CI	OR	95% CI
Injuries								
Workplace or commuting	626	3.86	1319	3.73	0.93	0.85-1.02	0.93	0.85-1.01
Workplace	425	2.62	847	2.39	0.90	0.79-1.03	0.89	0.78-1.02
Commuting	201	1.24	472	1.33	0.99	0.81-1.21	1.00	0.82-1.24
Types of injury								
Dislocations, sprains, and strains	261	1.61	538	1.52	0.92	0.77-1.08	0.92	0.78-1.09
Wounds and superficial injuries	227	1.40	433	1.22	0.83	0.69-1.00	0.81	0.67-0.98
Bone fractures	24	0.15	56	0.16	1.02	0.59-1.76	0.96	0.53-1.72
Causes of injury								
Sudden physical or mental strain (including noise and radiation)	99	0.61	171	0.48	0.85	0.65-1.11	0.85	0.64-1.12
Impact of a fixed surface or immobile cause (e.g., falling)	95	0.59	202	0.57	0.93	0.70-1.24	0.92	0.68-1.24
Cutting caused by a sharp or rough object	65	0.40	134	0.38	0.89	0.64-1.24	0.88	0.62-1.25
Physical violence (animal or human bite, kick, etc.)	75	0.46	133	0.38	0.78	0.55-1.11	0.76	0.53-1.09
A moving agent (including pressurized liquids and gases)	21	0.13	64	0.18	1.42	0.84-2.39	1.35	0.78-2.33
Compression or bruising	25	0.15	57	0.16	0.93	0.55-1.56	0.86	0.50-1.49
Common commuting injuries								
Falling, slipping, tripping, or overturning	157	0.97	360	1.02	0.98	0.78-1.23	1.00	0.79-1.27
Collision with a car, motorcycle, moped or bicycle	22	0.14	47	0.13	0.81	0.46-1.41	0.83	0.48-1.43

Model I: Adjusted for hierarchical structure of the data
Model II. Further adjusted for age, sex, number of days of work contract, number of night shifts, and the use of participatory shift scheduling software

Supplementary table S2: Difference in workplace and commuting injuries according to the use (no/yes) of the shift schedule evaluation tool limiting the follow-up to 2015-2017

Injury	All (30,084 non-users and 48,784 users)		Cities (5,834 non-users and 27,562 users)		Hospital districts (24,250 non-users and 21,222 users)	
	OR	95% CI	OR	95% CI	OR	95% CI
Injuries						
Workplace or commuting	0.95	0.87-1.04	0.84	0.72-0.98	1.00	0.90-1.10
Workplace	0.92	0.82-1.03	0.84	0.69-1.03	0.96	0.85-1.10
Commuting	1.02	0.87-1.20	0.80	0.63-1.03	1.09	0.90-1.32
Types of injury						
Dislocations, sprains, and strains	0.90	0.78-1.04	0.71	0.57-0.89	0.99	0.85-1.16
Wounds and superficial injuries	0.92	0.79-1.07	0.97	0.75-1.24	0.89	0.75-1.07
Bone fractures	1.12	0.74-1.69	0.84	0.44-1.58	1.27	0.78-2.08
Causes of injury						
Sudden physical or mental strain (including noise and radiation)	0.93	0.74-1.17	0.74	0.51-1.06	0.95	0.74-1.21
Impact of a fixed surface or immobile cause (e.g., falling)	0.98	0.77-1.24	0.88	0.58-1.33	0.99	0.77-1.26
Cutting caused by a sharp or rough object	1.03	0.78-1.37	1.53	0.92-2.53	0.90	0.65-1.24
Physical violence (animal or human bite, kick, etc.)	0.73	0.55-0.96	0.80	0.50-1.29	0.72	0.53-0.98
A moving agent (including pressurized liquids and gases)	1.33	0.87-2.04	1.15	0.60-2.21	1.45	0.92-2.27
Compression or bruising	1.11	0.66-1.87	0.82	0.41-1.61	1.43	0.78-2.61
Common commuting injuries						
Falling, slipping, tripping, or overturning	1.01	0.84-1.21	0.79	0.59-1.04	1.11	0.90-1.38
Collision with a car, motorcycle, moped or bicycle	1.10	0.69-1.76	0.94	0.47-1.87	1.12	0.63-2.01

Odds ratios adjusted for hierarchical structure of the data, age, sex, number of days of work contract, number of night shifts, and the use of participatory shift scheduling software
